# The MDCK variety pack: choosing the right strain

**DOI:** 10.1186/1471-2121-12-43

**Published:** 2011-10-07

**Authors:** Joseph D Dukes, Paul Whitley, Andrew D Chalmers

**Affiliations:** 1 Department of Biology and Biochemistry, Centre for Regenerative Medicine, University of Bath, Bath, UK

## Abstract

The MDCK cell line provides a tractable model for studying protein trafficking, polarity and junctions (tight, adherens, desmosome and gap) in epithelial cells. However, there are many different strains of MDCK cells available, including the parental line, MDCK I, MDCK II, MDCK.1, MDCK.2, superdome and supertube, making it difficult for new researchers to decide which strain to use. Furthermore, there is often inadequate reporting of strain types and where cells were obtained from in the literature. This review aims to provide new researchers with a guide to the different MDCK strains and a directory of where they can be obtained. We also hope to encourage experienced researchers to report the stain and origin of their MDCK cells.

## 

Madin-Darby Canine Kidney (MDCK) cells [1] are widely used as models for studying epithelia as they have clear apico-basolateral polarity, well defined cell junctions, a rapid growth rate, are suitable for confocal imaging and will polarise in 2D and 3D cell culture. In fact, when the term "MDCK" is searched for on PubMed over 5000 hits are generated [2] and reviews on epithelial cell trafficking, polarity and junctions in vertebrates rely heavily on experiments using MDCK cells [3-12]. In our experience we have certainly found MDCK cells to be a very tractable system [13]. Perhaps the only disadvantage to this cell line is its canine origin. This is important as commercially available antibodies are often raised against human or mouse sequences and may not cross-react well with canine proteins. Many siRNA reagents are also designed to human or mouse sequences.

A researcher new to epithelial cell polarity might wish to use such a useful cell line and would likely assume that obtaining it would be straightforward. However, the American Type Culture Collection (ATCC; [14]) catalogue has five different cell lines with "MDCK" within their titles (Table 1). Another popular supplier of cells, the European Collection of Cell Cultures (ECACC; [15]), has six different "MDCK" cell lines (Table 1). From these eleven MDCK strains, at least nine appear to be unique, including the parental MDCK line, MDCK I, MDCK II and confusingly two different lines termed MDCK.1 and MDCK.2.

**Table 1:** MDCK cell strains sold by three commonly used tissue culture vendors *

Supplier*	Strain	Description	Cat No
*ATCC *[14]	MDCK (NBL-2)	Parental cell line, heterogeneous population of cells.	CCL-34
	Supertube	Isolated from parental line, readily forms tubules in appropriate conditions.	CRL-2285
	Superdome	Isolated from parental line, readily forms large domes in appropriate conditions.	CRL-2286
	MDCK.1	Isolated from the parental cell line. Used in viral production/studies. **Not MDCK type I**.	CRL-2935
	MDCK.2	Isolated from the parental cell line. Used in viral production/studies. **Not MDCK type II**.	CRL-2936

*ECACC *[15]	MDCK (NBL-2)	Parental cell line, same as ATCC cat No. CCL-34.	85011435
	MDCK	Parental cell line, heterogeneous population of cells.	84121903
	MDCK I	Strain Isolated from a low passage parental cell line.	00062106
	MDCK II	Strain isolated from a high passage parental cell line.	00062107
	MDCK-Protein free	Strain modified to grow in protein-free medium. Results in growth of suspended clumps of cells.	02050101
	MDCK-SIAT1	Parental cell line cloned to give enhanced expression of 6-linked sialic acids. For virus research.	05071502

*JCRB *[33]	MDCK (NBL-2)	Parental cell line, same as ATCC cat No. CCL-34, unknown passage number.	IFO50071
	MDCK (NBL-2)	Parental cell line, same as ATCC cat No. CCL-34, passage number 55-58.	JCRB9029
	MDCK.P3	MDCK derivative, no serum required.	JCRB0717

*correct as of September 2011. This article focuses on the ATCC, ECACC and JCRB but other suppliers of cell lines are available, including the Deutsche Sammlung von Mikroorganismen und Zellkulturen (DSMZ) which does not currently supply MDCK strains

A quick literature search may not shed much light on which MDCK strain to use as, although many laboratories do provide accurate information, it is common to see researchers reporting "MDCK cells" with no reference to their strain or supplier. Others make statements which appear inaccurate, such as "MDCK II obtained from ATCC", when ATCC do not currently supply MDCK II cells (correct as of September 2011). Analysis of the methods sections of the top 50 hits for "MDCK" in PubMed would suggest between 30-50% of the published work fails to provide either the strain or supplier information, or provides apparently inaccurate information. Therefore, there is a clear problem in reporting strain identity and source within the literature which needs to be addressed.

The purpose of this article is to highlight the existence of multiple MDCK strains, to help new researchers find the correct strain and encourage improved reporting of MDCK cell line sources and strains. To achieve these aims the properties of the different MDCK strains and where they can be obtained from will be briefly reviewed. This information is summarised in Table 1. It should also be noted that clonal heterogeneity is not limited to MDCK cells. For example, there are at least six "HeLa" strains available from ATCC [14] and there has been clonal variability implied in many other cell lines, such as CHO (Konrad et al, 1977) and MCF-7 cells (Seibert et al 1983). Although this review focuses on MDCK cells, it should serve to raise awareness of possible variability in other mammalian cell lines.

### The parental MDCK (NBL-2) line

In 1958 Madin and Darby established two cell lines from bovine (MDBK) and ovine (MDOK) kidney [16]. They also derived the MDCK cell line from a seemingly normal adult female cocker spaniel but it appears they did not publish isolation of this line. However, MDCK cells were used to study viral infection [17], and subsequently characterised for the first time by Gaush *et al *in 1966 [1]. Then in the late 1970s/early 1980s the cell line started to become widely used for studying epithelial development and function [18].

The parental MDCK cell line, from which other strains of MDCK cells have been derived, is referred to as "NBL-2" and it was observed quite early on that low passage MDCK cells were not clonal. In fact they exhibited obvious heterogeneity in features including cell size and presence of an apical cilium [19]. This heterogeneity is important as experimental manipulation could select a particular population of cells [20], potentially complicating analysis of results. For example, a line with a distinctive 'semi-scattered' morphology and enhanced migration and invasion arose spontaneously from cultures of the parental MDCK cells [21]. This variant line was termed MDCK-1 but is not the same as MDCK I or MDCK.1, which are discussed below.

### MDCK I and MDCK II

Two other sub-types of MDCK cells were isolated from the parental strain [19,22,23] and designated type I and type II MDCK cells. MDCK I cells were isolated from low passage parental MDCK cells and display very high transepithelial resistance (TER) values (> 4000 Ω•cm^2^), indicating very "tight" junctions. MDCK II cells were obtained from higher passage MDCK cells and display much lower TER values (< 300 Ω•cm^2^) demonstrating "leaky" junctions [22]. This difference in TER is caused by differences in the composition of their tight junctions. Both strains express the tight junction proteins claudin-1, claudin-4, occludin and ZO-1 [24]. However, type II cells also express the pore-forming tight junction protein claudin-2 which may lower the TER of MDCK II cells [24].

The two strains show other differences in their epithelial junctions. Both strains form adherens junctions and desmosomes although MDCK I cells show much stronger staining for E-cadherin than MDCK II cells [25]. In contrast MDCK II cells show stronger basal desmosome staining [25]. Finally there is variability in gap junction formation [26]; type I cells have gap junctions, while type II cells do not form them unless forced to by expression of the gap junction proteins such as connexin 43 [27].

In addition to differences in junctions these strains are different in size, with MDCK II cells being larger and taller compared to the smaller flatter type I cells [23]. They also show distinct differences in their resident apical membrane proteins, Na-K-ATPase activity, expression of alkaline phosphatase and glycosphingolipid composition [22,23,28,29]. Interestingly, it should also be noted that ECACC states that MDCK I cells may display an unstable epithelial phenotype and that incomplete harvesting at confluence may select for a smooth muscle cell phenotype [15].

The parental, MDCK I and MDCK II strains have all been used to study epithelial cell polarity and junctions. MDCK II are the most commonly used strain and we would recommend them to researchers new to using MDCK cells, unless they have a specific reason to use one of the other strains. However, MDCK "type II like" cells have been isolated more than once and it is dangerous to assume these lines are the same. For example, two independently isolated clones (G and J) of MDCK type II cells, with similar ultrastructural organisation and TER, showed different modes of delivery of the Na/K-ATPase to the apical or basolateral surfaces [30]. This highlights the need for accurate reporting of strain origin in addition to strain type. The characteristics and suppliers of the parental, MDCK I and MDCK II strains are summarised in Table 2.

**Table 2:** The three MDCK strains that are most commonly used to study epithelial cell polarity

Strain	Details	Supplier*
*MDCK (NBL-2)*	Parental MDCK cell line from which type I and II are derived. A heterogeneous population of epithelial cells that will form a polarised monolayer. The heterogeneity makes it possible that manipulation might select a sub-population, potentially complicating analysis of experiments.	ATCC, ECACC, JCRB
*MDCK I*	Derived from low passage parental cell line (NBL-2). Type I cells display high TER values (> 1000 Ω•cm^2^), are negative for the tight junction claudin-2, and reported to display an adrenalin, vasopressin and prostoglandin E sensitive adenyl cyclise, unlike type II cells. Have gap junctions. The ECACC report that they may display an unstable epithelial phenotype.	ECACC (ATCC and JCRB do not supply)
*MDCK II*	Isolated from high passage parental cell line (NBL-2). Type II cells display low TER values (~100 Ω•cm^2^) and are positive for claudin-2. They are larger and taller than MDCK I cells and lack gap junctions unless modified. **MDCK II cells are the most commonly used MDCK strain and we would recommended them for most studies**. Not to be confused with MDCK.2 strain sold by ATCC.	ECACC (ATCC and JCRB do not supply)

*Correct as of September 2011. Other suppliers of cell lines are available however only ATCC, ECACC and JCRB have been considered here.

### Superdome and supertube

Under specific conditions MDCK cells can form fluid filled blister like structures called "domes" and occasionally long tubules termed "tubes". MDCK strains have been selected which form extensive domes (superdomes) or tubules (supertube) [31] and these lines can be obtained from the ATCC to study formation of these epithelial structures.

### MDCK cells as a model for infection

The other major use for MDCK cells is to study viral infection of cells and more recently in vaccine production [32]. A number of strains have been isolated to help with this research. These include "MDCK.1" and "MDCK.2" which are used mainly for the production and testing of viruses such as Influenza and should not be confused with MDCK I or MDCK II. The MDCK-SIAT1 line has also been isolated to aid infection studies.

In conclusion, the MDCK cell line provides an invaluable tool for understanding epithelial cell polarity, development and organisation. However, great care must be taken so that researchers are confident of the identity and source of the cells used and also report this information as accurately as possible.

## Authors' contributions

JDD wrote the first draft. ADC and PW edited the draft. All authors read and approved the final manuscript.
